# Concurrent Administration of Triazoles with Chemotherapeutic and/or Immunosuppressant Agents Known to Have Moderate-to-Severe Drug-Drug Interactions in Patients with Hematologic Malignancies Hospitalized for Invasive Aspergillosis

**DOI:** 10.1007/s11046-025-01043-4

**Published:** 2026-01-13

**Authors:** Thomas J. Walsh, Craig I. Coleman, Melissa D. Johnson, Belinda Lovelace, Barbara D. Alexander

**Affiliations:** 1 Departments of Medicine and Microbiology & Immunology, University of Maryland School of Medicine, Baltimore, MD USA; 2 Center for Innovative Therapeutics and Diagnostics, Richmond, VA USA; 3 Department of Pharmacy Practice, School of Pharmacy, University of Connecticut, 69 North Eagleville Road, Unit 3092, Storrs, CT 06269-3092 USA; 4 Infectious Diseases Division, Duke University, Durham, NC USA; 5 Health Economics and Outcomes Research, F2G, Inc., Princeton, NJ USA

**Keywords:** Triazoles, Drug interactions, Aspergillosis, Immunosuppressive agents, Chemotherapy, Hematologic neoplasms

## Abstract

**Background:**

Triazoles are widely used for treatment and prevention of invasive aspergillosis (IA) but can cause serious drug-drug interactions (DDIs) with chemotherapeutic (CT) and immunosuppressant (IS) agents via CYP3A4 inhibition. The frequency of triazole-CT or IS concurrent administration in hematologic malignancies (HM) patients newly admitted with IA is largely unknown.

**Methods:**

We studied US IQVIA claims including adults with ≥ 1 claim for an inpatient stay with a diagnosis code for IA from October 1, 2015-November 30, 2022 and evidence of systemic antifungal therapy for ≥ 3 days during the hospitalization. The cohort was limited to patients with ≥ 1 HM diagnosis code within 6 months prior to IA admission. Utilization of triazoles with CT and/or ISs known to have moderate-to-severe pharmacokinetic (PK) interactions was described.

**Results:**

Triazoles, predominantly isavuconazole (61.0%) and voriconazole (53.6%), were administered in 97.2% of 317 patients with IA. Of these, 241 (78.2%) received an interacting CT and/or IS. Potentially interacting agents administered with a triazole included corticosteroids (70.8%), calcineurin or mammalian target of rapamycin (mTOR) inhibitors (25.0%) (84.4% tacrolimus), alkylating agents (14.0%) (76.7% cyclophosphamide), venetoclax (9.7%), anthracyclines (6.2%), and vincristine (5.8%).

**Conclusions:**

Concurrent administration of triazole with potential PK interactions with CT or IS agents occurred in most HM patients admitted for IA. Choosing alternative antifungals, therapeutic drug monitoring of triazoles or selective ISs, and dosage adjustment of CT/IS agents may mitigate the risk of adverse DDIs. New antifungal agents without serious DDIs with CT and/or IS agents are needed for treatment of IA to reduce the risk of serious adverse events.

## Introduction

Invasive aspergillosis (IA), mostly caused by *Aspergillus fumigatus*, is estimated to occur annually in more than 2 million people worldwide [1]. Patients with hematologic malignancies (HMs), such as acute leukemias and lymphomas, are at a substantial risk of development of IA, particularly during periods of prolonged chemotherapy (CT)-induced neutropenia and immunosuppression [2–4]. The incidence of IA in these patients can range from 5.1–11.7%, with mortality rates often exceeding 50%, and reaching as much as 90% in hematopoietic cell transplant (HCT) recipients [5, 6].

Current guidelines recommend a mold-active triazole to be used as first-line for the prevention and treatment of IA for most patients [7, 8]. A key factor when considering mold-triazole use in patients with IA is the possibility of serious pharmacokinetic (PK) drug-drug interactions (DDIs). Many CT and immunosuppressant (IS) agents used to treat HMs undergo hepatic metabolism through cytochrome P450 (CYP) 3A4 isoenzyme or through drug transporters such as P-glycoprotein (P-gp) [9, 10]. Triazoles are moderate-to-strong inhibitors of the CYP3A4 and P-gp transporter molecules. Co-administration of triazoles with CT and IS agents that are substrates for CYP3A4 or P-gp may increase drug exposure and risk of serious adverse events (SAEs).

Data describing the real-world frequency of concurrently administering triazoles for IA with CT and/or IS agents known to have moderate-to-strong PK (CYP3A4 and/or P-gp) interactions are limited. We, therefore, sought to describe concomitant administration of triazoles with these agents in HM patients newly hospitalized for IA.

## Methods

This was a retrospective cohort study utilizing nationwide United States (US) claims data from the IQVIA New Data Warehouse [11]. This dataset includes deterministically linked data from professional fee and prescription claims data and IQVIA’s hospital charge data master (CDM) database for facilities across the US. These data contain ~ 1 billion professional fee claims per year (representing more than 870,000 practitioners per month) with diagnoses, procedures, and office-administered medications; approximately 1.6 billion retail or mail-order prescription claims representing dispensed prescriptions for about 85% of all pharmacies; and records from more than 450 hospitals, including inpatient and outpatient encounters within a facility with medication, procedure, diagnosis, and charge data for the entire stay.

Adults admitted for IA or developing IA during admission were identified based on International Classification of Diseases-10th-Revision (ICD-10) codes (B44.0, B44.1, B44.7) in the primary or non-primary hospital claim coding position between October 1, 2015 and November 30, 2022 (IA identification period). The first admission with IA during the patient identification period was utilized. The admission date was defined as the index date. To be included, patients had to have evidence of systemic antifungal therapy orders for ≥ 3 days during the hospitalization, link to the professional fees and pharmacy claims data sets during the study period, have activity denoted as ≥ 1 medical claim in the professional fee data set and ≥ 1 claim in the pharmacy claims data set during the baseline period, and exhibit pharmacy stability (i.e., consistent reporting of data from the pharmacy most frequently visited by the patient during each month of the baseline period). Patients with evidence of non-IA invasive fungal infection (e.g. invasive candidiasis) during the index hospitalization were excluded.

The study cohort was then limited to patients with HM. Data for the 6 months prior to the index date (baseline period) was used to assess patient baseline comorbidities, including HM defined as the presence of ≥ 1 claim associated with any type of encounter with an ICD-10 diagnosis code of C81-C96.

Triazole, CT and IS use was identified using National Drug Codes (NDCs) and Healthcare Common Procedure Coding System (HCPCS) codes starting day 0 of the hospital admission for IA. To be considered concurrently administered, the triazole and substrate CT and/or IS agents had to have overlapping usage based on inpatient medication use records and/or outpatient pharmacy claims data at some point between the day of admission (day 0) through last available follow-up (post-index period). The determination of CT or IS’s PK DDI severity with triazoles were determined based on published literature [9] and the National Aspergillosis Centre, Antifungal Trust’s Antifungal Interactions Database (available at: https://www.antifungalinteractions.org) [12, 13].

The concurrent administration of triazoles and CT and/or IS agents known to have moderate-to-severe PK interaction potential during the post-index period was assessed. Categorical data were reported as counts and percentages and continuous data as means ± standard deviations (SDs). Database management and statistical analysis was performed using SAS version 9.4 (SAS Inc., Cary, NC, USA).

All data were accessed in compliance with the Health Insurance Portability and Accountability Act (HIPAA). Institutional review board approval was not required for this retrospective analysis of de-identified secondary data. The data utilized in this study are available only by license through IQVIA.

## Results

Among the 317 patients with HM and IA, there were 699 CT/IS agents administered for a mean of ~ 2 agents per patient. Most of these patients (86.1%) were over 45 years of age and 63.4% were male (Table 1). The most common payer type was commercial (38.5%) and nearly two-thirds occurred in the West US Census region. Leukemias were the most common type of HM (82.0%), 55.5% of patients had neutropenia, and 34.7% had a history of bone marrow transplant (BMT) or HCT.

**Table 1:** Characteristics of hospitalized patients with hematologic malignancies and invasive aspergillosis

Characteristic	N = 317 n (%)
Age	
18–44 years	44 (13.9)
45–64 years	138 (43.5)
≥ 65 years	135 (42.6)
Male sex	201 (63.4)
Geographic Census region	
West	227 (71.6)
South	36 (11.4)
Midwest	31 (9.8)
Northeast	23 (7.3)
Payer type^#^	
Commercial	122 (38.5)
Medicare	111 (35.0)
Unknown/other	58 (18.3)
Medicaid	26 (8.2)
Year of index hospitalization	
2015*	7 (2.2)
2016	36 (11.4)
2017	70 (22.1)
2018	54 (17.0)
2019	76 (24.0)
2020	33 (10.4)
2021	21 (6.6)
2022*	20 (6.3)
Types of malignancy^†^	
Leukemia	260 (82.0)
Non-Hodgkin lymphoma	71 (22.4)
Comorbidities^∫^	
Neutropenia	176 (55.5)
Immunodeficiencies	124 (39.1)
BMT/HCT	110 (34.7)
Diabetes mellitus	92 (29.0)
Autoimmune conditions	7 (2.2)
Human immunodeficiency virus	5 (1.6)
End-stage kidney disease	3 (0.9)
Post-index triazole use^‡^	308 (97.2)

BMT/HCT = bone marrow transplantation or hematopoietic cell transplantation

^*^Partial year of data

^†^Not a complete list or mutually exclusive

^‡^Triazole use for ≥ 3 days during the hospitalization for invasive aspergillosis

^#^Commercial includes private and employer-sponsored health insurance plans; Medicare includes federal health insurance programs for adults ≥ 65 years and eligible younger individuals with disabilities or end-stage renal disease; Medicaid includes joint federal–state programs for low-income individuals; Unknown/other indicates payer type not documented or payer types outside the categories above

^**∫**^International Classification of Diseases, Tenth Revision codes for comorbidities included neutropenia (D70), immunodeficiencies (D80–D84, D86, D89), BMT/HCT (T86.0, T86.5, Z94.81, Z94.84), diabetes mellitus (E10-E11, E13, O24.0), autoimmune conditions (G35, G70, K90, L93, M05, M06, M08, M31.5, M32, M35.3), human immunodeficiency virus (B20, B97.35, O98.7, Z21), end-stage kidney disease (N18.6)

The mean duration of antifungal use, starting at the time of IA admission, was 146 ± 217 (median of 64) days. Triazoles were used in 308 of 317 (97.2%) patients over the course of treatment in the cohort (Table 2). The most prescribed triazoles were isavuconazole (61.0%) and voriconazole (53.6%), followed by posaconazole (27.6%), and itraconazole (1.6%). Amphotericin B and echinocandins were used in 35.3% and 60.6% of patients during the post-index period, including as monotherapy in 1.3% and 13.9% of patients, respectively.

**Table 2:** Triazole Use in Patients with Hematologic Malignancies Who Were Hospitalized with Invasive Aspergillosis

Triazole	N = 308 n (%)*	Strength of PK Interaction with CYP3A4^†^
Isavuconazole	188 (61.0)	+ +
Voriconazole	165 (53.6)	+ + +
Posaconazole	85 (27.6)	+ + +
Fluconazole	64 (20.8)	+ +
Itraconazole	5 (1.6)	+ + +

^*^More than one triazole may have been used per patient; thus, the column will total > 100%

^†^Strong severity (+ + +); moderate severity (+ +); mild severity ( +); no/minimal interaction (—)[9]

In total, 78.2% of patients who received triazoles also received a CT and/or IS agent with the potential for moderate-to-severe PK interactions (Table 3). The most frequent CT or IS agent administered during triazole therapy included corticosteroids (n = 218, 70.8%), calcineurin inhibitors (CNIs) or mammalian target of rapamycin (mTOR) inhibitors (n = 77, 25.0%) (of which 65/77 (84.4%) were tacrolimus), alkylating agents (n = 43, 14.0%) (33/43, 76.7% were cyclophosphamide), the B-cell lymphoma-2 inhibitor venetoclax (9.7%), anthracyclines (6.2%), and the vinca alkaloid vincristine (n = 18, 5.8%).

**Table 3:** Proportion of Patients Concomitantly Administered Triazoles with Chemotherapy and/or Immunosuppressants Known to Have Moderate-to-Severe Drug-Drug Interactions in Patients with Hematologic Malignancies and Invasive Aspergillosis

Chemotherapy or Immunosuppressant*	N = 308 n (%)^†^
At least one agent	241 (78.2)
Corticosteroids	218 (70.8)
Dexamethasone	153 (49.7)
Methylprednisolone	133 (43.2)
Calcineurin or mTOR inhibitors	77 (25.0)
Tacrolimus	65 (21.1)
Sirolimus	38 (12.3)
Cyclosporine	5 (1.6)
Alkylating agents	43 (14.0)
Cyclophosphamide	33 (10.7)
Busulfan	9 (2.9)
Ifosfamide	4 (1.3)
BCL-2 inhibitor (venetoclax)	30 (9.7)
Anthracyclines	19 (6.2)
Doxorubicin	7 (2.3)
Idarubicin	7 (2.3)
Daunorubicin	6 (1.9)
Vinca alkaloids (vincristine)	18 (5.8)
FLT3 inhibitors	7 (2.3)
Midostaurin	4 (1.3)
Gilteritinib	4 (1.3)
BCR-ABL inhibitors	6 (1.9)
Dasatinib	3 (1.0)
Ponatinib	2 (0.6)
Nilotinib	1 (0.3)
Imatinib	1 (0.3)
JAK inhibitors	5 (1.6)
Ruxolitinib	4 (1.3)
Fedratinib	1 (0.3)
Proteasome inhibitor (bortezomib)	5 (1.6)
BTK inhibitor (ibrutinib)	3 (1.0)
Immunomodulatory imide (lenalidomide)	2 (0.6)
HDAC inhibitor (romidepsin)	2 (0.6)
Hedgehog inhibitor (glasdegib)	1 (0.3)
Arsenic trioxide	1 (0.3)

^*^Chemotherapy and immunosuppressant agents with zero usage were not reported in the table

^**†**^More than one chemotherapy and/or immunosuppressant may have been used per patient; and others did not receive an interacting chemotherapy and/or immunosuppressant, so the column will not total to 100%

## Discussion

In this contemporary cohort of HM patients newly hospitalized with IA, triazoles were used in nearly all cases. Isavuconazole and voriconazole were used in more than 50% of HM patients, despite their known potential for DDIs with CT/IS agents. A potential triazole-CT/IS PK interaction known to be at least of moderate severity was present in more than 75% of patients. The most frequently concomitantly administered CTs included cyclophosphamide, venetoclax, and vincristine (range: 5.8–14.0% of cases). IS agents were also commonly used along with a triazole and included corticosteroids (70.8% of cases) and tacrolimus (84.4%).

CT or IS use in patients with HMs increases their risk for IA, particularly among patients with prolonged drug-induced neutropenia [2–4]. Current guidelines recommend mold-active triazoles as preferred antifungal agents for treatment of IA in most patients [7, 8]. All triazoles have PK interactions with most regimens used to treat HM, mainly through CYP3A4 and P-gp inhibition, albeit to varying degrees [9, 14, 15]. Itraconazole is a potent inhibitor of CYP3A4 and P-gp, posaconazole a potent inhibitor of CYP3A4, voriconazole a potent inhibitor of CYP3A4, CYP2C19 and CYP2C9, while isavuconazole is a moderate inhibitor of CYP3A4 and a mild inhibitor of P-gp, CYP2B6, organic cation transporter 2, and UDP-glucuronosyltransferase [14]. Isavuconazole was used more than other triazoles, possibly because of lesser inhibition of CYP3A4 and P-gp and despite higher acquisition cost compared to other azoles. Voriconazole is both an inhibitor and substrate of CYP2C19, CYP2C9, and CYP3A4 with the potential for a greater range of adverse DDIs than those of other triazoles [16–19].

While 78.2% of HM patients admitted for IA received a triazole known to have a moderate-to-severe PK DDI with their CT or IS agent(s), not all such interactions will lead to serious adverse events (SAEs) or will require pre-emptive dose adjustment. Increased vigilance may be sufficient in many cases.

Triazoles have different inhibitory potencies of CYP3A4 and P-gp [14, 15], while CT/IS agents may have wide or narrow therapeutic windows. The narrower the therapeutic window, the lower the exposure threshold needed for toxicity, which once surpassed, is more likely to result in an SAE [15]. Moreover, the types of toxicities associated with a particular CT or IS agents need to be considered. For example, in our study, we found triazole and venetoclax or vincristine were ordered during overlapping time frames in 9.7% and 5.8% of patients, respectively.

Clinically meaningful interactions such as with narrow therapeutic index drugs, such as calcineurin inhibitors (CNIs), tyrosine kinase inhibitors, and chemotherapeutic agents, such as vincristine and cyclophosphamide warrant dosage adjustment of the agent (CNI), the triazole, or complete avoidance of simultaneous administration. The FDA package insert for ruxolitinib warns against the concomitant use of CYP3A4 inhibitors during treatment of GVHD, myelofibrosis, and polycythemia vera (PCV), which may cause neutropenia and thrombocytopenia. In order to prevent these events, some programs reduce the dosage of fluconazole or posaconazole for antifungal prophylaxis or definitive therapy. A thoughtful patient-centered assessment throughout the course of cancer therapy, immunomodulation, and HCT transplant of the potential serious DDIs assures dosage adjustment or change to another antifungal agent reduces the probability of adverse events.

All triazoles have at least a moderate level PK interaction with venetoclax (considered a severe interaction with itraconazole, posaconazole and voriconazole, and a moderate interaction with isavuconazole via CYP3A4 inhibition). When a triazole is concomitantly administered with venetoclax, patients experience a higher rate of tumor lysis syndrome (particularly, during the initiation and dose-titration phases) [16–19]. Similarly, vincristine has the potential for a moderate-to-severe PK interaction with all triazoles due to CYP3A4 inhibition and P-gp, and when concurrently administered, is associated with a higher rate of severe neurotoxicity, [14, 20–22]. In both examples, if these CT agents must be concomitantly administered with a triazole, several approaches may be implemented including substantial dose reductions of the CT agent [9, 15, 17], withholding of the triazole for an interval before and after administration of the CT [21], or utilization of alternative non-triazole antifungal agents. While DDI/physiological base and pharmacokinetic analysis suggests that isavuconazole is less likely to induce vincristine neuropathy [23], prescribing information for isavuconazole states that the two medications should not be co-administered owing to an estimated two-fold increased exposure of the vinca alkaloid [24].

Another SAE that HM patients are at an increased risk due to concurrent administration of a triazole and certain CT/IS agents includes QTc prolongation and the possible subsequent development of life-threatening arrhythmia Torsades de Pointes (TdP). Specific CT agents to treat HM that are associated with important QTc prolongation include a wide range of compounds: arsenic trioxide, cyclophosphamide (alkylating agent), gilteritinib and midostaurin (FLT3 inhibitors), glasdegib (Hedgehog inhibitor), lenalidomide (immunomodulatory amide), and nilotinib (BCR-ABL inhibitor) [25, 26]. When triazoles inhibit these agents' metabolism through CYP3A4, the increased drug exposure to the CT agent can further prolong QTc interval, placing patients at higher risk for TdP and sudden cardiac death. Importantly, both posaconazole and voriconazole also cause QTc prolongation [9, 14, 15] which may further exacerbate the QTc prolongation caused by their concurrent administration with CT/IS agents. Isavuconazole, which shortens the QTc interval [27], has been used in lieu of other triazoles when required for administration with QTc-prolonging CT/IS agents.

The demographic data in this study reveal that approximately 43% of patients were 65 years of age. Elderly patients have increased risk for development and more serious adverse outcomes of DDIs in relation to altered hepatic, renal, and metabolic status that affects the pharmacokinetics and pharmacodynamics of drugs, especially those with narrow therapeutic indices in the elderly population compared to those of younger cohorts. [28, 29]. Given that 29% of patients had concomitant diabetes mellitus, diabetes-related renal and hepatic dysfunction, may further add to the limitations of age-dependent declines. Depending upon the age, co-morbidities, possible drug combinations, as well as hepatic and renal functions, further dosage adjustment may be needed to prevent and adverse DDI.

Clinicians should be trained to anticipate and recognize drug interactions in these high-risk patients, and while guidance is available on how to proactively avoid such drug interactions [8, 9], clinicians should be prepared to individualize plans for avoiding and managing potential PK DDIs [9, 14, 15]. Clinicians may preferentially choose triazoles with comparatively lower PK DDI potential, such as isavuconazole, to perform therapeutic drug monitoring (TDM) of both the triazole and CT/IS, dose reduce the interacting CT/IS agent based upon recommendations from prescribing information, practice guidelines, or other reliable sources, or avoid certain azole-CT/IS agent interactions (e.g., a triazole + vincristine) altogether by using amphotericin B or an echinocandin to treat IA. In fact, in our study, we observed substantial use of these antifungal agents, including monotherapy. Finally, patients should always be monitored closely for signs or symptoms of toxicities, including, neuropathy, tumor lysis syndrome, hepatotoxicity, QTc prolongation, and cardiac arrhythmias [14].

Our study has several limitations. First, IA was identified by ICD-10 diagnosis codes, but neither a formal diagnosis nor the severity of IA could be verified due to the lack of available clinical and laboratory data in our data set (i.e., potential for misclassification bias) [4, 7, 8, 11, 30]. To optimize our study’s specificity in capturing invasive disease, we restricted out inclusion to the presence of codes B44.0, B44.1, B44; notably excluding B44.8x (other forms of aspergillosis) and B44.9 (aspergillosis, unspecified). This methodology likely decreased the sample size of our study but increased the probability that we captured true cases of invasive aspergillosis. Similarly, the absence of clinical and/or laboratory data meant we were unable to provide more specific data on comorbidities such as the severity of neutropenia or end-stage kidney disease. Second, while we used both published literature and an antifungal DDI database [9, 12–15], expert opinion or guidelines on the clinical relevance of DDIs between specific triazoles and CT or IS agents will no doubt vary in routine practice. While triazoles and CT/IS agents were frequently used concomitantly for IA, due to a lack of laboratory data the dataset did not permit an evaluation of the simultaneity of dosing adjustment to avoid an SAE. The dataset also was unable to assess the frequency of treatment-related triazole-induced adverse events. The diverse nature or potential adverse events make capturing them in a claims-based dataset complex and unreliable. Thirdly, medication usage could have occurred outside of IQVIA's data partners [11]. Such utilization would not be captured in our data set and could result in missed triazole and/or CT/IS medication usage instances. Next, as data for this study spanned 2015–2022, changes in routine practice patterns for IA and/or HM management may have changed. Finally, our population of HM patients with IA captured from the IQVIA’s New Data Warehouse may not be entirely representative of all such patients in the US. This, in part, may explain the geographic distribution of HM patients with IA seen in our study, though prior data also suggests IA infection is highest in the West and low in the Northeast [31].

In summary, concurrently administered triazoles and CT/IS agents with potential for moderate-to-severe PK interactions occurred in most patients with HM who were hospitalized for IA. These interactions have the potential to cause SAEs if not anticipated. Prevention or mitigation of triazole DDIs may consume already limited healthcare resources to perform dosage adjustments and TDM. The higher rate of isavuconazole observed in this population compared to other triazoles may be a signal of physicians’ recognition of the need to reduce the risk of DDI-related SAEs and toxicity. Choosing alternative antifungals, TDM of triazoles, alteration of dosing intervals of triazoles, and dosage adjustment of CT/IS agents should all be considered and evaluated in future studies of routine clinical practice. Antifungal agents, especially oral formulations, without or with a lesser potential for serious DDIs with CT and/or IS agents are needed for treatment of IA.

## Data Availability

The data utilized in this study are available only by license through IQVIA.
